# Epidemiology of *Haemophilus influenzae* in children on Lombok Island, Indonesia

**DOI:** 10.1099/acmi.0.000609.v4

**Published:** 2023-08-02

**Authors:** Nina Dwi Putri, Korrie Salsabila, Ari Prayitno, Shindy Claudya Aprianti, Wisiva Tofriska Paramaiswari, Made Ananda Krisna, Dodi Safari

**Affiliations:** ^1^​ Department of Child Health, Dr Cipto Mangunkusumo National Central Hospital, Faculty of Medicine, University of Indonesia, Jakarta, Indonesia; ^2^​ Eijkman Research Centre for Molecular Biology, National Research and Innovation Agency, Cibinong, West Java, Indonesia; ^†^​ Present address: Biology Department and Nuffield Department of Clinical Medicine, University of Oxford, Oxford, UK

**Keywords:** *Haemophilus influenzae*, *Haemophilus influenzae* serotype b, Lombok Island, Indonesia, non-typeable *H. influenzae*

## Abstract

The *Haemophilus influenzae* serotype b (Hib) conjugate vaccine routine immunization programme has been implemented for almost a decade; however, there is limited surveillance of *H. influenzae* carriage rates in the Indonesian population. *H. influenzae* was isolated from nasopharyngeal (NP) swab specimens of healthy children on Lombok Island, West Nusa Tenggara Province, Indonesia from 2018 to 2019. Serotyping was performed using quantitative polymerase chain reaction. We identified *H. influenzae* in 40 of the 96 (41.6 %) NP swab specimens. We identified 39 non-typeable *H. influenzae* (NTHi) isolates and 1 Hib isolate.

## Data Summary

The authors confirm all supporting data, code and protocols have been provided within the article or through supplementary data files.

## Introduction

*Haemophilus influenzae* is a Gram-negative coccobacillus that causes non-invasive diseases, such as otitis media and sinusitis, and invasive diseases, such as pneumonia and meningitis. Based on capsule polysaccharide expression, this pleomorphic bacterium is categorized as non-capsulated or non-typeable (non-typeable *H. influenzae *; NTHi) and capsulated or typeable, which includes six serotypes (a–f) [1, 2]. During the pre-vaccine era, serotype b (Hib) accounted for 95 % of all invasive diseases due to *H. influenzae* and was the most common cause of bacterial meningitis in children <5 years of age, with 83 % of cases occurring in children <2 years old. The burden of Hib disease remains significant, especially among children <5 years, even though the Hib vaccine has been introduced in routine infant immunization programmes in many countries [1, 3]. Additionally, an increasing number of non-Hib invasive diseases have been consistently reported in the post-Hib vaccine era [4]. The Hib vaccine, as part of the pentavalent vaccine, was integrated into a routine immunization programme in Indonesia in 2013, but post-vaccination surveillance of *H. influenzae* cases is still very limited [5, 6].

*H. influenzae* is primarily transmitted through direct contact with respiratory droplets from nasopharyngeal carriers [7]. Nasopharyngeal *H. influenzae* carriers are considered the only reservoirs and transmission vehicles for invasive diseases. Reports showed that the rate of *H. influenzae* carriers is approximately 20 % in infants and >50 % in children aged 5–6 [8]. This study investigated the prevalence of *H. influenzae* carriers in children in Lombok, several years after implementation of a routine Hib immunization programme. Additionally, the results of the current study likely reflect similar epidemiological trends in most regions in the country, which correlate with the public health and socioeconomic issues in Lombok Island.

## Methods

Archived nasopharyngeal (NP) swab specimens from a previous prospective cohort pneumococcal carriage study on Lombok Island, West Nusa Tenggara, Indonesia, conducted between March 2018 and June 2019, were used [9]. The study enrolled healthy infants (2 months old) who were brought to the primary care facility for routine vaccination and were randomized into two groups: pneumococcal vaccine (PCV)-vaccinated group and the non-PCV-vaccinated group (control group). The two groups were matched based on age and home location. All recruited children were healthy when the NP swab specimens were obtained [9]. In the current study, we randomly sampled 96 of 233 archived NP swab specimens from both the vaccine (64 specimens) and control (32 specimens) groups, which were obtained 6 months after the PCV vaccine booster was administered to the vaccine group. The sample size was calculated based on the sample size formula for estimating a single proportion with an approximate power of >0.95.

NP swab specimen-inoculated skim milk, tryptone, glucose and glycerol (STGG) medium was thawed, and 100 µl was transferred onto a supplemented chocolate agar plate (5 % sheep blood and vitox/isovitalex) with 20 U ml^−1^ bacitracin (CAPwB). Plates were incubated at 37 °C with 5 % CO_2_ for 18–24 h. Suspect *H. influenzae* colonies were characterized as oxidase-positive; non-haemolytic; opaque to grey colour; large, round, convex colonies; creamy texture with pungent indole smell [10]. They appeared as Gram-negative coccobacilli with pleomorphic morphology. The X and V factor test was performed since *H. influenzae* only grows around the XV disc [10, 11]. A single colony-forming unit of confirmed *H. influenzae* isolate was restreaked, harvested and then stored in STGG medium at −80 °C as a stock isolate. All suspected *H. influenzae* colonies were confirmed by detecting the *hpd* gene and serotyped utilizing a real-time polymerase chain reaction. The gene targets for serotypes a, b, c, d, e and f are *acsB*, *bcsB*, *ccsD*, *dcsE*, *ecsH* and *bexD*, respectively, as previously described [11].

The laboratory results were summarized as the total prevalence of carriage and the distribution of serotypes among the positive results. We also reviewed relevant provenance data collected using a questionnaire developed in a previous study, which included the following variables: sex, nutritional status, number of people in the household and cigarette smoke exposure [9].

## Results and Discussion

We isolated *H. influenzae* from 40 of the 96 NP swab specimens [41.6 %, 95 % confidence interval (CI): (31.7 %, 52.2 %)] (Table 1) . Based on polymerase chain reaction-based serotyping, 39 isolates were NTHi and only 1 was Hib. Among the positive specimens, 23 (57.5 %) and 17 (42.5 %) were from males and females, respectively (Table S1). We also found that 69 % of participants had been exposed to cigarette smoke in their households.

**Table 1.** Prevalence of *H. influenzae* carriage among Indonesian children and the adult population

Population	Year	Region	*n*	Prevalence (%)	Serotype (%)							Ref.
					a	b	c	d	e	f	NTHi	
Healthy children <5	2018	Lombok Island	96	41.6 95 % CI (31.7 %, 52.2 %)	0	2.5	0	0	0	0	97.5	This study
Schoolchildren with AOM aged 6 to 12 years	2018	Purwokerto	122	69.7	0	3.5	0	0	0	1.2	95.3	[6]
Healthy children aged	2016	Bandung, Lombok Island, Padang	302	27.5 95 % CI (22.5 %, 32.9 %)	nt	0	nt	nt	nt	nt	nt	[5]
12–24 months												
Children with HIV infection	2012	Jakarta	90	18	0	0	0	0	0	0	100	[11]
(mean age 69 months)												
Adults with HIV infection	2012	Jakarta	200	9	0	0	0	0	0	0	100	[11]
(mean age 35 years)												
Healthy children (0 to 2 years of age)	1997*	Lombok Island	484	32	2.9	64.7	2.9	8.8	11.8	8.8	nd	[12]

nt, not tested; nd, no data; AOM, acute otitis media.

*In this study, only 34 out of 155 *H. influenzae* isolates were serotyped.

The prevalence of *H. influenzae* carriage in this study was higher than that reported in a previous pre-Hib vaccination era study (32 % of 484 subjects) in Lombok in 1997 (Table 1). However, the prevalence of Hib in our study (2.5 %, 1/40) was lower than that reported by this same Lombok 1997 study (14 %, 22/155) [12]. Another study conducted in Bandung, Lombok and Padang in 2016 also found an *H. influenzae* carriage rate of 27.5 % in 302 subjects, which was lower than that in the current study, even though no Hib was detected, probably because of the implementation of a routine Hib immunization programme. A report on *H. influenzae* carriage rate among children with HIV infection in Jakarta found a significantly lower rate of 18 % [11]. Recently, the prevalence of *H. influenzae* was reported to be 69.7 % (85/122) among schoolchildren with acute otitis media in Banyumas Regency, Central Java, Indonesia, with 3.5 and 1.2 % of the isolates being *H. influenzae* serotypes b and f, respectively [6] (Table 1).

The impact of the routine Hib immunization programme, which has been implemented for almost a decade, is reflected in the reduced rate of Hib carriage. However, the overall increased prevalence of *H. influenzae* carriage found in the current study, with predominantly NTHi, remains a concern. NTHi is the major upper respiratory commensal that occasionally causes both non-invasive diseases, such as acute otitis media, sinusitis and conjunctivitis, and invasive diseases (e.g. meningitis and septicaemia) [13]. A convincing body of evidence has shown a shift in invasive cases caused by non-type b *H. influenzae *, especially NTHi, in many countries [4, 5]. Therefore, NTHi, as a common group in carriers, can be considered to be a pool of potentially pathogenic strains that can lead to invasive diseases in susceptible individuals. Unfortunately, in many developing countries such as Indonesia, it is difficult to detect the *H. influenzae* serotype that causes invasive diseases, or even to detect the bacterium at all, as there are only a few laboratories capable of isolating and identifying *H. influenzae *. The evidence of increasing *H. influenzae* carriage rates should provoke consideration for routine active surveillance of *H. influenzae* diseases and improve laboratories’ capacity to identify and characterize them based on serotypes. Additionally, based on the serotype distribution results of our study, it can be predicted that NTHi will be the dominant group underlying invasive *H. influenzae* infection

## Supplementary Data

Supplementary material 1
